# Per-Arnt-Sim Kinase (PASK): An Emerging Regulator of Mammalian Glucose and Lipid Metabolism

**DOI:** 10.3390/nu7095347

**Published:** 2015-09-07

**Authors:** Dan-dan Zhang, Ji-gang Zhang, Yu-zhu Wang, Ying Liu, Gao-lin Liu, Xiao-yu Li

**Affiliations:** Department of Clinical Pharmacy, Shanghai General Hospital, School of medicine, Shanghai Jiaotong University, No.100 Haining Road, Shanghai 200025, China; E-Mails: RainbowDan0513@hotmail.com (D.-D.Z.); grissomzhang@hotmail.com (J.-G.Z.); yzwyy0506@126.com (Y.-Z.W.); liuying2e@163.com (Y.L.); gaolinliu@aliyun.com (G.-L.L.)

**Keywords:** Per-Arnt-Sim kinase (PASK), metabolic syndrome (MetS), glucose and lipid metabolism, protein regulation

## Abstract

Per-Arnt-Sim Kinase (PASK) is an evolutionarily-conserved nutrient-responsive protein kinase that regulates lipid and glucose metabolism, mitochondrial respiration, phosphorylation, and gene expression. Recent data suggests that mammalian PAS kinase is involved in glucose metabolism and acts on pancreatic islet α/β cells and glycogen synthase (GS), affecting insulin secretion and blood glucose levels. In addition, PASK knockout mice (PASK-/-) are protected from obesity, liver triglyceride accumulation, and insulin resistance when fed a high-fat diet, implying that PASK may be a new target for metabolic syndrome (MetS) treatment as well as the cellular nutrients and energy sensors—adenosine monophosphate (AMP)-activated protein kinase (AMPK) and the targets of rapamycin (m-TOR). In this review, we will briefly summarize the regulation of PASK on mammalian glucose and lipid metabolism and its possible mechanism, and further explore the potential targets for MetS therapy.

## 1. Introduction

Metabolic Syndrome (MetS) is characterized by glucose intolerance, insulin resistance (IR), central obesity, diabetes, hypertension, and dyslipidemia, all of which increase the risk of cardiovascular diseases, diabetes, and chronic kidney diseases, as well as increased mortality [1,2]. It was estimated that MetS is present in 71% of adult hypertensive subjects and affects approximately 22% of US adults and 4.2% of US adolescents according to the modified National Cholesterol Educational System Program Adult Treatment Panel III (NCEP ATP III [3]). Therefore, understanding the etiology of MetS is of great importance to explore potential targets for the therapy of metabolic diseases. Currently, metabolic disorders caused by cellular lipid and glucose imbalance and dysregulation, islet dysfunction, inflammation, and other genetic or environmental factors were proposed as the underlying causes of MetS. Adenosine monophosphate-activated protein kinase (AMPK) and mammalian targets of rapamycin (mTOR), as cellular nutrient and energy sensors, have been closely associated with metabolic syndrome-related diseases and might be effective targets for MetS prevention and therapy [4].

Similarly, as a nutrient sensor, Per-Arnt-Sim kinase (PASK) is an emerging regulator in lipid and glucose metabolism [5,6] and may be a new target for treatment of MetS. PASK is a canonical serine/threonine kinase that contains a PAS domain. It was predominantly found in HeLa cell nuclear extracts by mass spectrometry [7,8] and with slightly higher expression in brain [9], liver [10], prostate [11], and testis [12]. As a signal integrator [12], PASK exists in yeast, drosophila, and mammals and is highly conserved with several orthologs [13]. PASK also regulates lipid and glucose metabolism, mitochondrial respiration, phosphorylation, and gene expression [5,14,15,16]. Currently, we observed that there are limited original articles focusing on PASK [5,14,17] and most of the previous papers [5,18,19] focus on the metabolic regulation of PASK in yeast. Therefore, in this review, we would like to summarize the regulation of PASK in mammals, identify how it regulates lipid and glucose metabolism, and explore a potential target for treatment of MetS.

### 1.1. PAS Kinase Structure and Function

**Figure 1:**
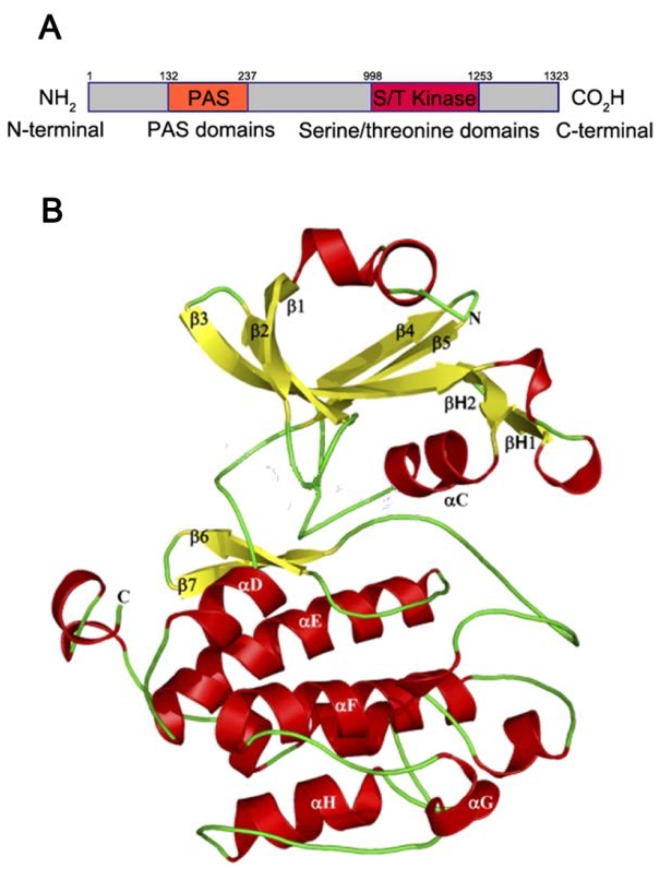
(**A**) Schematic diagram of the domain architecture of Per-Arnt-Sim Kinase (PASK). PASK usually contains a Per-Arnt-Sim (PAS) domain (orange square) and kinase domain (red square). The *N*-terminus of the PAS domain is described as “NH_2_” while the *C*-terminus of kinase domain is indicated to be “CO_2_H”; (**B**) The crystal structure of the PAS domain of PASK. The structure of PAS domain of PASK is usually an α/β helix, that is folded with several α helices depicted in red and surrounded by a five-stranded antiparallel β-sheets in yellow, composing a structurally-conserved core. The loops are shown in green (Fα/FG loop), and the *N* and *C* termini of the kinase structure are indicated as N and C [22].

PASK possesses an *N*-terminal PAS domain and a *C*-terminal kinase domain [20] (Figure 1A). The *N*-terminal PAS domain, which belongs to a large member of the super family of PAS domains [21], displays low sequence conservation and high functional diversity. According to Gardner *et al.* [20], who determined a high resolution nuclear magnetic resonance (NMR) structure of the human PAS kinase (hPASK) PAS domain, the structure of hPASK PAS domain usually contains an α/β helix [5,20] that is composed of several α helices (Cα, Dα, Eα, and Fα) surrounded by a 5-stranded (Aβ, Bβ, Gβ, Hβ, and Iβ) anti-parallel beta-sheet (Figure 1B [22]). Meanwhile, the long and dynamic loop segment (Fα/FG loop) [20] is also important for signal transduction purposes by facilitating interactions with the *C*-terminal kinase domain and PAS domain. As with other PAS domains, the PAS domain of hPASK binds small organic molecules within its structurally conserved [21] and hydrophobic [15] α/β helix core, allowing hPASK to sense changes from a variety of cellular and environmental conditions and to serve as sensory modules for a variety of intracellular cues, including light, oxygen, the redox state, and various metabolites. In addition, the *C*-terminus of hPASK contains a canonical serine/threonine kinase domain that regulates energy utilization via phosphorylation of protein substrates [15,22], including transcriptional activators, guanylate cyclases, phosphodiesterases, ion channels, and kinases. Therefore, it was indicated that PASK might play a pivotal role in coordinating energy sensing with metabolic control due to the two primary domains and their respective functions [15,22].

### 1.2. PAS Kinase Activation

**Figure 2:**
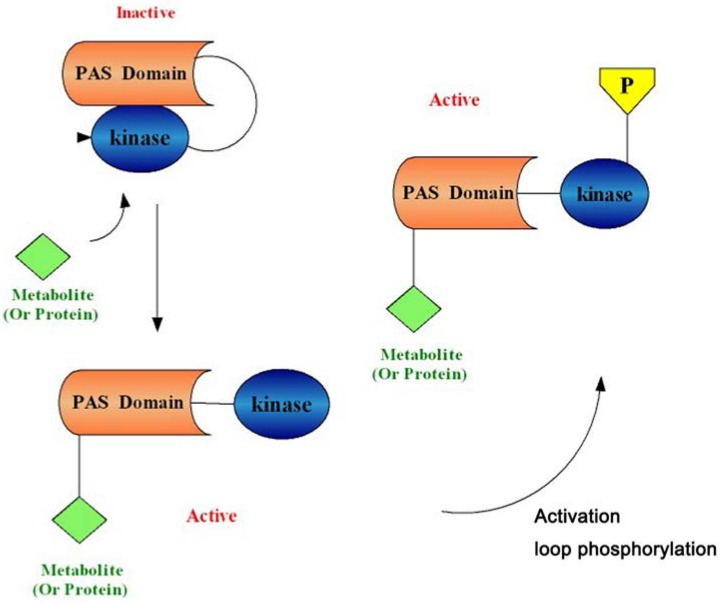
A model for Per-Arnt-Sim Kinase (PASK) activation. The Per-Arnt-Sim (PAS) domain binds to and inhibits the kinase domain. A metabolite or protein (green square) activates PAS kinase by binding to the PAS domain and relieving PAS domain inhibition. Activation loop phosphorylation (yellow pentagon) is not required for basal PASK activation, but often plays a role in substrate binding and catalysis. PASK is then competent to phosphorylate substrates involved in glucose partitioning and translation to elicit the appropriate physiological response [5,20,22].

The PAS domain binds to the kinase domain and inhibits basic PASK functions. This binding and inhibition may be prevented by the association of a small metabolite or protein with the PAS domain, thus interfering with the binding between the PAS domain and the kinase domain to activate the enzyme [5,20] (Figure 2). In addition, it has been reported that most protein kinases require phosphorylation of serine/threonine or tyrosine residues within the activation loop (or P loop) of the kinase domains to achieve full activation, and PASK possesses a phosphorylatable threonine residue in the canonical activation loop site [7]. However, Kikani *et al.* [22] found that the kinase domain of PASK adopts an active conformation and has catalytic activity *in vivo* and *in vitro* in the absence of activation loop phosphorylation, implying that phosphorylation may not be required for the basal activity of the kinase. The main reason for not requiring phosphorylation might be the presence of an alanine at position 1161, which is occupied by lysine in many protein kinases that require activation loop phosphorylation. Although PASK, as a eukaryotic protein kinase (RD protein kinase), could successfully bypass the required loop phosphorylation for basal activation, it cannot be neglected that loop phosphorylation could modulate PASK activity in specific contexts or regulate its activity in a substrate-specific or non-canonical manner, and it could even play a structural role in stabilizing a catalytically active conformation [15,22].

## 2. The Function and Mechanism of PASK in Mammalian Glucose and Lipid Metabolism

### 2.1. The Regulation of PASK in Mammalian Glucose Metabolism

Sugar is an organic compound that has a chemical nature that can be categorized into two types: Polyhydroxyaldehyde and polyhydroxyketone. It exists naturally as glucose (Glc) or glycogen (Gn), which are vital to mammalian glucose metabolism and their metabolism frequently provides the energy needed for oxidation. In mammals, Glc and Gn can be used in a variety of different processes including glucose consumption (catabolism) for the production of usable energy (adenosine triphosphate (ATP)) and glucose homeostasis, in which glucose is stored in the carbohydrate form as glycogen or converted to lipid or protein for storage [23]. Once the balance of these two processes is broken, MetS, such as diabetes, obesity or other metabolic disorders, can easily occur. PASK is known to be a physiological regulator of glucose metabolism, functioning in pancreatic islet cells or glycogen synthase (GS) and regulating glucagon and insulin secretion (Table 1). Importantly, it can significantly influence glucose and glycogen transformation and blood glucose level [17,24].

**Table 1:** The regulation of PASK in mammalian glucose metabolism.

Name	Expression Level	Biological Function	Reference
Insulin	Up-regulated	Promotes the absorption of glucose from the blood to skeletal muscles and fat tissue; causes fat to be stored.	[25,26,27]
Glucagon	Down-regulated	Elevates the concentration of glucose in the blood by promoting gluconeogenesis and glycogenolysis.	[17]
Glycogen synthase (GS)	Down-regulated	A key enzyme in glycogenesis; involves in converting glucose to glycogen.	[26,28]
GSK-3β	Down-regulated	Phosphorylates and inactivates its downstream target GS; active in a number of central intracellular signaling pathways (cellular proliferation, migration, inflammation, immune responses, glucose homeostasis, and apoptosis)	[29]

#### 2.1.1. PASK and Pancreatic α Cells

Pancreatic α cells are endocrine cells that secrete glucagon and elevate blood glucose by promoting glycogenolysis and gluconeogenesis. However, excessive glucagon secretion is also one of the causes of obesity and hyperglycemia [30]. Therefore, the proper way in which to reduce excessive glucagon secretion and balance the interchange between glycogen and glucose has gradually become an issue. When cultured pancreatic alpha-TC1-9 cells were extracted from mice *in vitro* and siRNA was used to silence PASK by way of PASK mRNA degradation, the results showed an elevated level of glucose-stimulated glucagon secretion [31]. The reason for this observation might be an autonomous action of the cell on cellular activities or changes in membrane excitability mediated by the release of beta cell-derived cytokines (the level of unbound Ca^2+^, cyclic adenosine monophosphate (cAMP), *etc.* [31]). In any case, PASK inhibits glucagon secretion and decreases the blood glucose level; therefore, it is theoretically possible to treat such metabolic diseases as insulin resistance, inadequate insulin secretion, and type II diabetes by effectively regulating glucagon and insulin secretion [14]. Although further clinical experiments based on connections between PASK and pancreatic α cells require more research, it is still a matter worthy of consideration.

#### 2.1.2. PASK and Pancreatic β Cells

Pancreatic β cells promote insulin secretion in mammals, which elevates glycogenesis and generates glycogen, which is stored in the liver and muscles. Francesca *et al.* [25] reported that PASK-modulated glucose-stimulated insulin secretion in a CD1 mouse model, HEK293T cells, and 18 young onset diabetic probands (three probands: diabetes diagnosed before age 30, no requirement of exogenous insulin in the two first years and an autosomal dominant inheritance of type 2 diabetes) of French families. Furthermore, when studied in Min6 cells [26], it was surprising that PASK-silenced Min6 cells showed down-regulated expression of insulin-relevant genes and reduced insulin secretion, implying that PASK participated in insulin secretion and might be related to the direct phosphorylation of the transcription factor pancreatic duodenal homeobox-1 (PDX-1) (a necessary substance for insulin production [32]). In addition, to determine the specific mechanism, Da Silva Xavier *et al.* [26] cultured mouse pancreatic β cells that were micro-injected with purified wild-type PASK *in vitro* and subsequently found that the expression of preproinsulin and PDX-1 were both elevated and that insulin secretion was increased. This may be related to the inhibition of PASK in response to chronic exposure to palmitate [27] (a substance that decreases PDX-1 activation and hinders insulin secretion). PASK expression or over-expression ameliorated the effects of palmitate on PDX-1 activation so that activated PDX-1 could normally elevate glucose-induced preproinsulin expression and form insulin in the Golgi apparatus [33]. This analysis was consistent with the conclusion above [26], which raises the possibility that PASK is a key mediator affecting insulin secretion in pancreatic β cells through palmitate. Meanwhile, when Da Silva Xavier *et al.* [26] assessed single MIN6 cells injected with unmodified preproinsulin, PDX-1 promoter and purified wild-type PASK, they found highly increased PDX-1 expression and elevated insulin production after incubation with medium containing 30 mM (*versus* 3 mM) glucose [34], suggesting that the up-regulation of PASK on insulin only worked under high levels of glucose (low glucose and insulin stored in cells was invalid).

Intriguingly, there are reciprocal interactions between PASK and glucose. Da Silva Xavier *et al.* [26] revealed that the expression of PASK in ordinary MIN6 cells transfected with hPASK or wild-type PASK presented a significant increase in 30 mM glucose medium, while the cells micro-injected with an anti-PASK antibody completely abrogated the transcriptional response to 30 mM glucose. In addition, the expression of the PASK in MIN6 cells cultured with 30 mM glucose increased by approximately 50% compared to the MIN6 cells cultured in 3 mM with no fluctuant and enhanced PASK expression [26], indicating that PASK expression was positively affected by a high glucose level. Because PASK regulates insulin secretion in β cells, researchers have begun to study the connection between PASK and type II diabetes [31,35]. 

#### 2.1.3. PASK and Glycogen Synthase

GS is the rate-limiting enzyme of glycogenesis, regulating the interchange between glucose and glycogen. Wilson *et al.* [28] proposed that mammalian PASK could efficiently phosphorylate and inactivate GS at Ser-640 [36] in both rabbit muscle and HEK293 cells. In addition, when exploring the effects of PASK on blood glucose with MIN6 cells, Da Silva Xavier *et al.* [26] also found the connection between PASK and GS: PASK acted on pancreatic β cells, phosphorylating and inactivating glycogen synthase kinase 3β (GSK-3β), which efficiently alleviated the PDX-1 degradation [29]. As mentioned above [33], PASK certainly increased insulin secretion as it enhanced PDX-1 stability, reducing blood glucose. Interestingly, the results produced by the co-purification suggested that PASK could down-regulate glycogenesis in the extra cellular matrix (ECM) [5], but the mechanism was still uncertain. Considering that PASK efficiently inhibits GS activation accompanied with decreased glycogenesis and elevated blood glucose levels, it may significantly regulate glucose metabolism. Thus, the screen and application of PASK inhibitors that can appropriately regulate GS expression in cells may become a new target of treatment of diabetes and other metabolic diseases.

### 2.2. The Regulation of PASK in Mammalian Lipid Metabolism

Lipids are important substances that store energy for oxidation and metabolism. As the main cause of imbalanced lipid metabolism, excessive lipid accumulation in liver has been implicated in the development of MetS, such as diabetes, obesity, hepatic adipose infiltration, and unpredicted morbidity. Therefore, maintaining a balance between lipid synthesis and catabolism is of great importance. Notably, because Katschinski *et al.* [10] first revealed the regulative function of PASK on lipid metabolism from a PASK knockout mouse model (PASK-/-), subsequent researchers [25,33,37,38] have paid more attention to PASK-/-. When they established the PASK-/- model, they found that PASK-/- mice fed a high-fat diet could be successfully protected from obesity owing to increased mitochondrial respiration accompanied with alteration on expression of a series of proteins or receptors [39] involved in lipid metabolism (Table 2), which might provide evidence for researching the new treatments of the metabolic diseases caused by lipid metabolic disorders.

**Table 2:** The regulation of deficient PASK in mammalian lipid metabolism.

Name	Expression Level	Biological Function	Reference
AMPK	Up-regulated	Stimulates hepatic fatty acid oxidation and ketogenesis; inhibits cholesterol synthesis, lipogenesis, and triglyceride synthesis; inhibits adipocyte lipolysis and lipogenesis; stimulates skeletal muscle fatty acid oxidation and muscle glucose uptake, modulates insulin secretion.	[9,40]
SREBP-1c	Down-regulated	Promotes cholesterol biosynthesis and uptake; stimulates fatty acid biosynthesis.	[37]
FAS	Down-regulated	An enzyme complex responsible for fatty acid biosynthesis; catalyzes the synthesis of palmitate from acetyl-CoA and malonyl-CoA.	[6,33]
CD36/FAT	Down-regulated	Functions in long-chain fatty acid uptake and signaling; promotes sterile inflammation.	[6,33]
SCD1	Down-regulated	A key enzyme in fatty acid metabolism, catalyzes a rate-limiting step in the synthesis of unsaturated fatty acids.	[6,33]
PPARγ	Down-regulated	Regulates fatty acid storage and glucose metabolism; stimulates lipid uptake and adipogenesis.	[6,33]

AMPK, adenosine monophosphate-activated protein kinase; SREBP-1c, sterol regulatory element binding protein-1c; FAS, Fatty acid synthase; CD36/FAT, cluster of differentiation 36/fatty acid translocase; SCD1, stearoyl-CoA desaturase 1; PPARγ, peroxisome proliferators-activated receptors γ.

#### 2.2.1. PASK and AMPK/SREBP-1

AMPK is a class of protein kinase that is widespread in eukaryotic cells and acts as “the regulator of cell energy”, regulating energy metabolism in the liver [41], heart [42] and skeletal muscle [43]. Once activated by an upstream protein kinase termed adenosine monophosphate-activated protein kinase kinase (AMPKK), AMPK works with its downstream proteins to modulate nutrient metabolism and energy balance. Furthermore, when AMPK was activated, AMPK also enhanced the biosynthesis of mitochondria and phosphorylation of lipid metabolism enzymes (sterol regulatory element binding protein 1 (SREBP-1), acetyl-CoA carboxylase (ACC), malonyl-CoA, GS, *etc.*), inhibiting activities such as fatty acid synthesis, isoprenoid synthesis, triglyceride synthesis, phospholipid synthesis or glycogenesis. AMPK alternatively enhances the oxidative metabolism of fatty acid and glycerol trimesters and reduces fat accumulation to balance the body’s fat content.

As a nutrient-sensing regulator [14], PASK plays an important role in AMPK expression and function. Initially, the presence of PASK in the nerve cells of the hypothalamus (N2A) is necessary for AMPK activation [9]. In contrast, a study of PASK-/- mice fed a high-fat diet found that AMPK expression was increased in liver extracts, and the signal conduction of AMPK’s responding in liver cells was also increased and was accompanied by the down-regulated activation of its direct downstream target, ACC [40]. This was perhaps one of the reasons why PASK-/- mice fed high-fat diet could be successfully protected from obesity, peripheral insulin resistance, and hepatic steatosis [6,33]. In addition, it has been reported [44] that AMPK participates in mitochondrial respiration, increasing the metabolic rate and energy expenditure. Therefore, Hao *et al.* [33] measured the CO_2_ consumption and O_2_ production in normal mice and PASK-/- mice that were both fed high-fat diets. The results showed that the metabolic rate, metabolism of mitochondria and mitochondrial respiration in PASK-/- mice were all similarly increased and accompanied by a hyperactive metabolism and increased ATP production. Nevertheless, according to an electron micrograph of the soleus muscle and subsequent quantitative analysis, the number of mitochondria was not changed and citrate synthase (a marker of density of mitochondria) [45] was almost equal in both the normal mice and PASK-/- mice [33], indicating that deficient PASK enhances mitochondrial respiration through the AMPK pathway; however, it does not change the number of mitochondria and the internal environments. Substrate (both glucose and palmitate) oxidation and the cellular ATP content were significantly increased in PASK-silenced L6 myoblast cells [33], similar to the trend shown *in vivo*. Taken together, this is perhaps another reason why PASK-/- mice fed a high-fat diet could be successfully protected from obesity through the AMPK pathway.

As a transcription factor, the SREBP-1 is a principal regulator of lipogenesis, which is also one of the downstream proteins of AMPK [46]. Rutter *et al*. [37] reported that PASK was required for the proteolytic maturation of SREBP-1c *in vitro* and *in vivo*. They also proved that a PASK inhibitor (Bio-E1115) could significantly decrease serum and liver triglyceride concentrations, partially reverse insulin resistance owing to the downregulated expression of SREBP-1c and its target genes. Similar results were obtained in PASK-/- mice model and PASK-silenced HepG_2_ cells.

#### 2.2.2. PASK and other Enzymes or Receptors Involved in Lipid Metabolism

It has been reported [5,6] that a PASK deficiency can also affect the expression or activation of enzymes related to lipid synthesis or fatty acid synthesis. From the PASK-/- mice model, Hao *et al.* [33] observed a decreased expression of several genes, including the fatty acid transporter fatty acid translocase (CD36), stearoyl-CoA desaturase 1 (SCD1), and the lipid-responsive nuclear hormone receptor peroxisome proliferators-activated receptors γ (PPARγ). The mechanism of this down-regulation by PASK deletion awaits further investigation, however, it is widely believed, as shown by Huai-Xiang Hao *et al.* [6], that the pregnane X receptor (PXR) [47] may have contributed to the decreased level of cytochrome P450 3A11 (CYP3A11, a target gene of PXR) in PASK deficient liver [33].

This also signified that PASK is involved in lipid metabolism, affecting the expression of PPAR or other genes/synthases, and that PASK deficiency can effectively reduce the accumulation of fat and inhibit the increased weight caused by uptaking substantial high-fat diets.

## 3. Future Directions

MetS is also associated with non-alcoholic fatty liver disease (NAFLD) [48,49,50] exhibiting NAFL (non-alcoholic fatty liver), NASH (non-alcoholic steatohepatitis), and cirrhosis. The main pathological factors leading to NAFLD might be abnormal lipid accumulation, oxidative stress, and metabolic disorders of glucose and lipids. More importantly, besides metabolic disorder, it was known that the elevated expression of inflammatory cytokines is one of the major factors accelerating the process from NAFL to NASH, even to cirrhosis. Hotamisligil *et al.* [51] implied that insulin action, metabolic disease clusters, obesity, insulin resistance, and type II diabetes are closely associated with chronic inflammation characterized by abnormal cytokine production.

**Figure 3:**
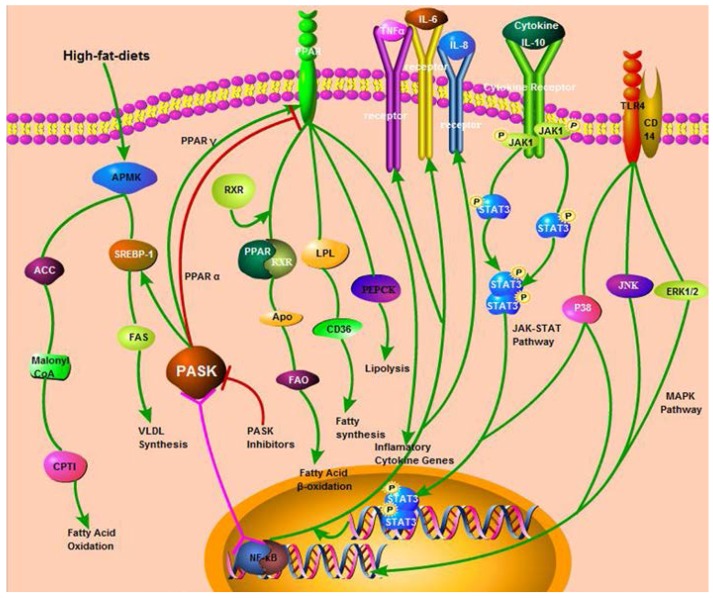
PASK signaling pathways with their downstream targets and direct biological Regulation, PASK has several functions on Lipid metabolism and inflammation. The green line represents active function, the negative effect is in red, and the pink line stands for the uncertain relationship that still needs to be further proven.

Considering that PPAR could down-regulate the expression of tumor necrosis factor α (TNF-α), interleukine-1 beta (IL-1β) and interleukine-6 (IL-6) through the mitogen-activated protein kinases (MAPK), janus kinase-signal transducers and activators of transcription (JAK-STAT) or nuclear factor kappa-B (NF-κB) pathways [52,53], we speculate that PASK may also play a potential role in the expression of inflammatory cytokines based on the interaction between PPAR and PASK (Figure 3). Although it is still unknown, it will become a new research area applied in clinical therapy if PASK can down-regulate the expression of inflammation cytokines, inhibiting the deterioration from NAFL to NASH or cirrhosis, which should be addressed in future research.

## 4. Conclusions

As shown by a wide range of studies, PASK acts on pancreatic islet α/β cells, regulating insulin secretion and blood glucose levels. Meanwhile, PASK could inhibit GS in extracellular matrix (ECM), decreasing glycogenesis. In addition, PASK deficiency provides protection from high fat diet-induced obesity and insulin resistance by affecting the expression or activation of enzymes and receptors involved in fat synthesis and increasing mitochondrial respiration, implying that PASK may become a potential target for the treatment of MetS. Further research on the effects and mechanisms of PASK is required to determine its importance as a target for treating metabolic diseases.
